# Inhibition of the Self-Assembly of Aβ and of Tau by Polyphenols: Mechanistic Studies

**DOI:** 10.3390/molecules24122316

**Published:** 2019-06-22

**Authors:** Qiuchen Zheng, Micheal T. Kebede, Merc M. Kemeh, Saadman Islam, Bethany Lee, Stuart D. Bleck, Liliana A. Wurfl, Noel D. Lazo

**Affiliations:** Carlson School of Chemistry and Biochemistry, Clark University, 950 Main Street, Worcester, MA 01610, USA; qzheng@clarku.edu (Q.Z.); mkebede@clarku.edu (M.T.K.); mkemeh@clarku.edu (M.M.K.); sislam@clarku.edu (S.I.); blee@clarku.edu (B.L.); sbleck@clarku.edu (S.D.B.); lwurfl@clarku.edu (L.A.W.)

**Keywords:** Alzheimer′s disease, amyloid-β self-assembly, tau self-assembly, tau hyperphosphorylation, amyloid assemblies, neurofibrillary tangles, polyphenols

## Abstract

The amyloid-β (Aβ) peptide and tau protein are thought to play key neuropathogenic roles in Alzheimer’s disease (AD). Both Aβ and tau self-assemble to form the two major pathological hallmarks of AD: amyloid plaques and neurofibrillary tangles, respectively. In this review, we show that naturally occurring polyphenols abundant in fruits, vegetables, red wine, and tea possess the ability to target pathways associated with the formation of assemblies of Aβ and tau. Polyphenols modulate the enzymatic processing of the amyloid-β precursor protein and inhibit toxic Aβ oligomerization by enhancing the clearance of Aβ42 monomer, modulating monomer–monomer interactions and remodeling oligomers to non-toxic forms. Additionally, polyphenols modulate tau hyperphosphorylation and inhibit tau β-sheet formation. The anti-Aβ-self-assembly and anti-tau-self-assembly effects of polyphenols increase their potential as preventive or therapeutic agents against AD, a complex disease that involves many pathological mechanisms.

## 1. Introduction

Alzheimer’s disease (AD) is the leading cause of dementia worldwide. According to the World Alzheimer Report 2018, about 50,000,000 people in the world have dementia, and about two thirds, or more than 30,000,000, have AD. Clinically, AD is characterized by progressive cognitive impairment that inevitably leads to severe dementia, a stage marked by acute loss of almost all cognitive functions. Biochemically and biophysically at the cellular level, AD is characterized by extracellular amyloid plaques and intraneuronal neurofibrillary tangles (NFTs).

Amyloid plaques, found mostly in the isocortex, are composed primarily of amyloid-β (Aβ) peptides, which are produced from the sequential cleavage of the amyloid-β precursor protein (AβPP) by β- and γ-secretases [1]. The predominant forms of Aβ contain 40 or 42 amino acids, commonly identified as Aβ40 and Aβ42, respectively. Aβ42 is more hydrophobic, has a higher propensity to form insoluble fibrils, and thus, is more abundant in plaques than Aβ40. The formation of fibrils is hierarchical in nature, indicated schematically as follows (Scheme 1):

Biophysical studies have demonstrated that the oligomers, protofibrils and fibrils of Aβ, contain increasing β-sheet contents [2]. In vitro toxicity studies have demonstrated that the Aβ assemblies are neurotoxic, but there is now general agreement that oligomers, Aβ42 oligomers to be precise, are the most pathogenic form of Aβ [3,4].

NFTs, found in the cytosol of neurons, are composed of paired helical filaments (PHFs), which are twisted, fibrous, β-sheet-containing assemblies of the tau protein. Tau is a microtubule-associated protein that plays a role in the stabilization of neuronal microtubules and in the regulation of axonal transport and outgrowth [5]. It is a soluble, predominantly disordered protein [6] that self-assembles to form oligomers and fibrils. The latter assembly aggregates further to form PHFs and NFTs (Scheme 2).

Given that the common characteristic of Aβ and tau in AD is abnormal self-assembly, we hypothesize that molecules that inhibit the self-assembly of Aβ *and* tau are attractive therapeutics against AD. Naturally occurring molecules called polyphenols have been shown to significantly modulate the self-assembly of Aβ and tau. This article identifies such molecules and discusses the proposed mechanisms behind the inhibition of self-assembly.

## 2. Chemical Properties of Polyphenols of Relevance to This Review

Polyphenols are small molecules that contain one or more phenolic rings. They are classified into curcuminoids, flavonoids, lignans, phenolic acids, stilbenes, and tannins [7]. Table 1 presents the chemical structures and common sources of the polyphenols included in this review.

**Figure 1:**
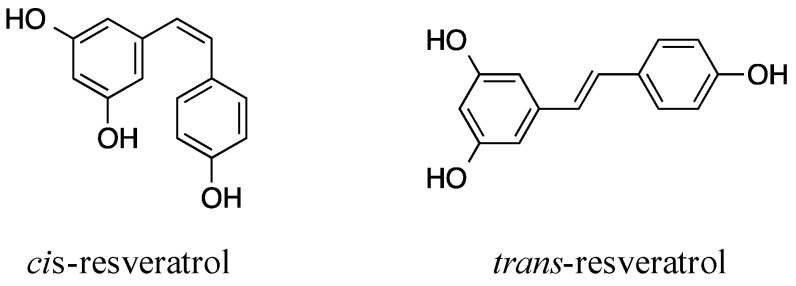
Isomers of resveratrol. Trans-resveratrol is more stable than cis-resveratrol and accounts for the beneficial effects of the polyphenol.

Resveratrol (RES) is a stilbene that has anticancer [8], antioxidant [9] and neuroprotective [10] properties. RES has two isomers, cis- and trans-resveratrol (Figure 1), but the latter is more stable and responsible for the properties of the polyphenol [11]. RES is rapidly metabolized and therefore has low bioavailability [12]. Nonetheless, both RES and its major metabolites are able to cross the blood-brain barrier (BBB) [13] and thus, these molecules possess the potential to accumulate at pharmacologically relevant concentrations in the brain.

Rosmarinic acid (RA), a phenolic acid, is an ester of caffeic acid and 3,4-dihydroxyphenyllactic acid. RA has neuroprotective, antioxidant and anti-inflammatory effects, as discussed in a recent review [14]. It is quite soluble in water, and thus organic solvents are not required for in vitro studies of the effects of the polyphenol (e.g., [15]). However, the high solubility of RA in aqueous solvents means that its ability to cross the BBB is low. If true and if in vitro and in vivo studies indicate that the potential of RA for the prevention and/or cure of AD is high, then addressing the delivery of RA across the BBB will be important.

**Figure 2:**
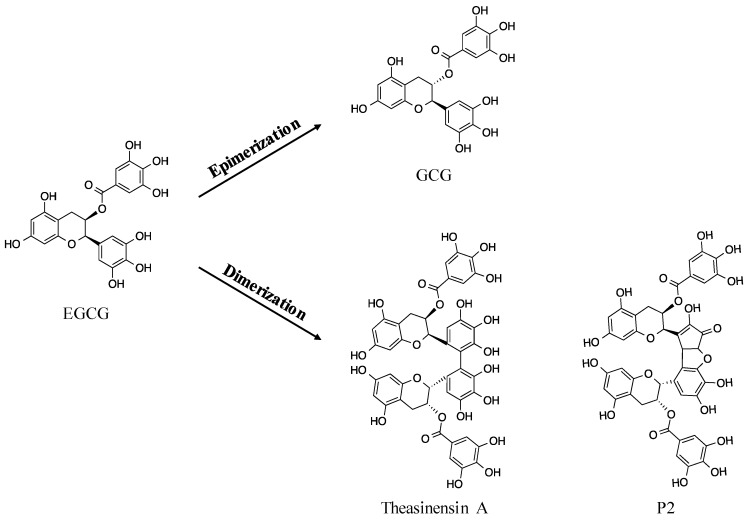
Under common experimental conditions, epigallocatechin-3-gallate (EGCG) forms gallocatechin-3-gallate (GCG) through epimerization, and theasinensin A and P2 through oxidation-induced dimerization.

Epigallocatechin-3-gallate (EGCG) is a flavonoid and is the most abundant catechin in green tea made from the leaves of *Camellia sinensis*. A recent review highlights the anticancer, anticardiovascular, neuroprotective, anti-oxidant, anti-obesity, antidiabetic and anti-allergic effects of EGCG [16]. However, EGCG has low bioavailability and thus, efforts are underway to develop nanoformulations of EGCG designed to prevent the rapid metabolism of the molecule (e.g., [17]). EGCG is slightly soluble in water but becomes more soluble in ethanol and similar solvents. Under cell culture conditions, EGCG undergoes oxidation to form digallate dimers, theasinensin A and P2, and epimerization to form gallocatechin-3-gallate (GCG) (Figure 2) [18]. Reaction rates are affected by concentration of EGCG, pH, temperature, and the partial pressure of O_2_. Thus, mechanistic studies of the biological effects of EGCG should take into consideration its stability.

Curcumin (CUR) is a curcuminoid that possesses antioxidant, anti-inflammatory, anticarcinogenic and neuroprotective effects as reviewed recently [19]. It is a linear diphenylheptanoid containing two o-methoxy phenolic rings linked by a seven-carbon chain (Table 1). Because it is lipophilic, CUR is able to cross the BBB, as shown in a number of studies using laboratory rodents (e.g., [20]). At 37 °C, CUR degrades in solutions with pH ≥ 7; however, in acidic pH, its half-life increases by two orders of magnitude [21].

Gallic acid (GA), aka 3,4,5-trihydroxybenzoic acid, is a phenolic acid that has strong anticancer properties [22]. In humans, GA is absorbed more compared to other polyphenols [23]. It is converted into other molecules primarily by glucuronidation and methylation, with 4-*O*-methylgallic acid being one of the key methyl derivatives in the body [24].

Quercetin (QUE), a flavonoid, is a potent antioxidant found in many fruits, vegetables and food products such as apples, onions, spinach, broccoli, kale, and tea. As such, it is routinely consumed in the diet. The bioavailability of QUE in humans is subject to significant variation between individuals [25].

RES, CUR, GA and QUE are sparingly soluble in water (Table 1) but in our studies using RES and CUR, we prepared concentrated stock solutions in ethanol followed by dilution with the desired buffer [26,27,28]. A caveat of this approach is that it can lead to precipitation of the polyphenol. However, this was not observed in our studies and more importantly, we were able to ascribe differences in outcomes of test and control experiments to the effect of the polyphenol on the biophysical properties of either Aβ42 [26] or amylin [27,28].

## 3. Polyphenols Inhibit Aβ Self-Assembly

### 3.1. Modulation of Aβ Production

Because Aβ self-assembly is driven by an increase in the concentration of Aβ monomer, reducing Aβ monomer levels is an attractive strategy for inhibition. One way to accomplish this is through modulation of Aβ production by increasing the activity of α-secretase and inhibiting β-secretase.

**Figure 3:**
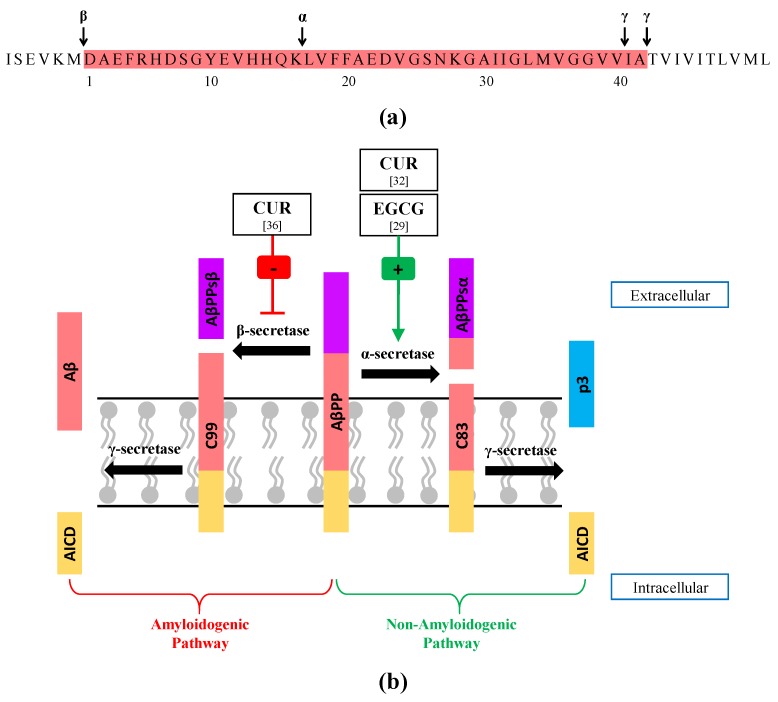
Enzymatic processing of amyloid-β precursor protein (AβPP). (**a**) Cleavage within the amyloid-β (Aβ) sequence (i.e., at the peptide bond between Lys16 and Leu17) by α-secretase precludes Aβ production while sequential cleavages first by β-secretase, and then by γ-secretase at the sites indicated produce Aβ40 and Aβ42. (**b**) Amyloidogenic and non-amyloidogenic pathways and their products. Curcumin modulates the amyloidogenic pathway by inhibiting β-secretase. EGCG and curcumin facilitate the non-amyloidogenic pathway by enhancing the activity of α-secretase.

The processing of AβPP is divided into two pathways: non-amyloidogenic and amyloidogenic. The non-amyloidogenic pathway, which precludes Aβ production, starts with the cleavage of the Lys16–Leu17 peptide bond within the Aβ domain (Figure 3a) by α-secretase, releasing AβPPs to the extracellular space (Figure 3b). The C-terminal fragment C83 is processed by γ-secretase, releasing p3 to the extracellular space and the amyloid-β precursor protein intracellular domain (AICD) to the cytoplasm. The amyloidogenic pathway begins with the β-secretase cleavage of the Met–Asp1 peptide bond (Figure 3a), releasing AβPPsβ into the extracellular space (Figure 3b). Processing of the C-terminal fragment C99 by γ-secretase releases Aβ to the extracellular space and AICD to the cytoplasm. Several studies have shown that polyphenols modulate the production of Aβ in two ways: enhancement of the α-secretase mediated cleavage of the Lys16–Leu17 peptide bond by EGCG and CUR, and inhibition of β-secretase by CUR (Figure 3b).

#### 3.1.1. Enhancement of α-Secretase Activity

Rezai-Zadeh et al. demonstrated that EGCG treatment of murine N2a cells transfected with human AβPP modified by the Swedish mutation (K670N/M671L), and primary neuronal cells derived from AD Tg2576 mice results in a significant decrease in Aβ production [29]. Additionally, they found that the production of C83 and AβPPs are increased in these cells after EGCG treatment, consistent with the enhancement of α-secretase activity. To validate their findings in vivo, Rezai-Zadeh et al. intraperitoneally or intracerebroventricularly injected EGCG into AD Tg2576 mice and found reduced Aβ levels associated with the enhancement of the nonamyloidogenic α-secretase mediated pathway [29]. Subsequently, Obregon et al. showed that activation of ADAM10, one of several members of the a disintegrin and metalloprotease (ADAM) family implicated as putative α-secretase candidates, is required for EGCG promotion of α-secretase cleavage of AβPP [30]. This result led to the conclusion that ADAM10 is an attractive pharmacotherapeutic target for the treatment of cerebral amyloidosis in AD. However, ADAM10 has a wide range of substrates, and enhancing its activity may lead to unwanted side effects [31]. We surmise, however, that the health benefits of EGCG [16], including anti-oxidant and neuroprotective effects, coupled with its effect on α-secretase activity, are worth consideration in the development of therapeutic approaches for AD.

Narasingapa et al. treated HEK293 cells overexpressing AβPP with CUR and its derivatives [32]. They reported that CUR enhances the activity of α-secretase but when CUR is conjugated at the two phenolic positions with hydrophobic amino acids including isoleucine, phenylalanine or valine, the activity of α-secretase is increased even more. The mechanism behind this effect is not known.

#### 3.1.2. Inhibition of β-Secretase

The β-secretase BACE (β-site amyloid-precursor-protein-cleaving enzyme) is the rate-limiting enzyme in the production of Aβ [33]. Several studies have shown that BACE inhibition is a potential strategy for AD therapeutics. For example, Keskin et al. applied histochemistry, in vivo Ca^2+^ imaging and behavioral analyses in APP23xPS45 transgenic mice, and demonstrated that BACE inhibition is beneficial to all levels of impairment in the AD mouse model, i.e., inhibition rescued hyperactivity of neurons, impairment of long-range circuitry, and memory defects [34]. However, clinical trials of verubecestat, an orally administered BACE-1 inhibitor, have failed [35].

Wang et al. used a FRET-based enzyme assay to show that curcuminoids present in turmeric inhibit the activity of β-secretase [36]. The curcuminoids arranged in the order of increasing IC_50′_s are: bisdemethoxycurcumin < demethoxycurcumin < curcumin. This result indicates that the absence of methoxy groups in the phenyl rings of CUR (Table 1) increases the inhibition of β-secretase. The abilities of EGCG [37] and resveratrol [38] to inhibit β-secretase have been investigated, and it was shown that neither one modulate the activity of the enzyme.

Because EGCG does not modulate the activity of β-secretase [37], Mori et al. tested the combination of EGCG and ferulic acid (FA), a β-secretase modulator [39], in APP/PS1 mice [40] which express human AβPP bearing the Swedish mutation, and PSEN1 with the L166P mutation that increase the Aβ42/Aβ40 concentration ratio [41]. They showed that the combination had consequential advantages over single treatment with either EGCG or FA. In particular, reversal of cognitive impairment in tests of learning and memory, amelioration of cerebral amyloidosis, and reduction of Aβ production were observed [41]. We hypothesize that other combinations of naturally occuring compounds (e.g., EGCG and CUR) may lead to similar or even better results.

### 3.2. Polyphenols Inhibit Toxic Aβ Oligomerization

**Figure 4:**
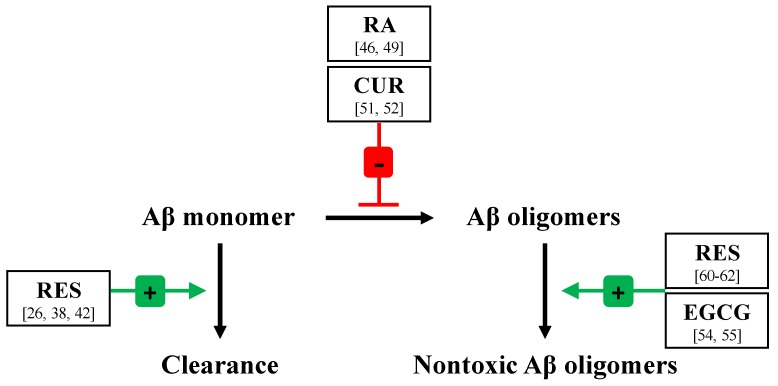
Modulation of the formation of toxic Aβ oligomers by polyphenols. Resveratrol enhances the clearance of Aβ monomer. Rosmarinic acid and curcumin directly inhibit oligomerization by interfering with peptide–peptide interactions while resveratrol and EGCG remodel toxic Aβ oligomers to nontoxic assemblies.

Figure 4 presents three ways by which polyphenols target the pathway of toxic Aβ oligomerization. RES enhances the clearance of Aβ monomer, CUR and RA inhibit Aβ oligomerization, and RES and EGCG remodel Aβ oligomers to nontoxic forms.

#### 3.2.1. Enhancement of Aβ Monomer Clearance

Several laboratories have shown that RES facilitates the clearance of Aβ in neuronal cells [38,42]. The mechanism for the clearance of Aβ is not well understood but several mechanisms have been proposed. Vingtdeux and coworkers hypothesized that the clearance may involve the activation of AMP-activated protein kinase which in turn inhibits the mammalian target of rapamycin (mTOR) resulting in the initiation of autophagy and lysosomal clearance of Aβ [42]. Marambaud and coworkers proposed that RES promotes the clearance of Aβ40 and Aβ42 by the proteasome [38]. Others have shown that RES upregulates the expression of insulin-degrading enzyme (IDE) in the hippocampus [43]. More recently, we showed that RES sustains the activity of IDE towards Aβ42 monomer in two ways [26]. First, the number of initial cleavage sites is increased. Using limited proteolysis monitored by mass spectrometry, we showed that the initial cleavages in the absence of RES occur in the central hydrophobic cluster (CHC), i.e., at the peptide bonds between Phe19 and Phe20 and between Phe20 and Ala21. In the presence of RES, a third initial cleavage site occurs at the peptide bond between Lys28 and Gly29, which is found in the putative turn region of Aβ [44]. This has biophysical significance in that hydrophobic interactions between the CHC and the AIIGL segment of Aβ, which are hypothesized to stabilize in part the structure of a disease-relevant Aβ42 fibril [45], are prevented. Second, RES facilitates further IDE-dependent degradation of the primary fragments of Aβ42 to smaller fragments. This is important because primary C-terminal fragments can aggregate and seed self-assembly of Aβ peptides. Together, our results suggest that the combination of RES and IDE holds promise for therapeutic and/or preventive strategies for AD.

#### 3.2.2. Modulation of Aβ Monomer–Aβ Monomer Contacts

Hamaguchi et al. [46] showed that oral administration of RA prevented the development of Aβ neuropathology in AD Tg2576 mice which express human AβPP modified by the Swedish mutation (K670N/M671L) associated with increased production of Aβ [47]. Analysis of Aβ in the soluble fractions of the brain indicated that RA inhibits the formation of A11-positive Aβ oligomers [46]. This result appears to be relevant because A11-positive oligomers correlate with cognitive deficits in AD transgenic mice models [48]. Ono et al. used several biophysical techniques including atomic force microscopy, circular dichroism, nuclear magnetic resonance (NMR), and photo-induced cross-linking of unmodified proteins and showed that RA inhibits the oligomerization of Aβ40 and Aβ42 [49]. This suggests that the polyphenol modulates contacts between Aβ monomers. Because Aβ oligomers impair synaptic plasticity and memory by inhibiting long term potentiation (LTP) and enhancing long term depression (LTD) [50], Ono et al. used LTP and LTD assays of hippocampal slices from C57BL/6 mice and showed that RA diminished Aβ oligomer-induced synaptic toxicities [49].

**Figure 5:**
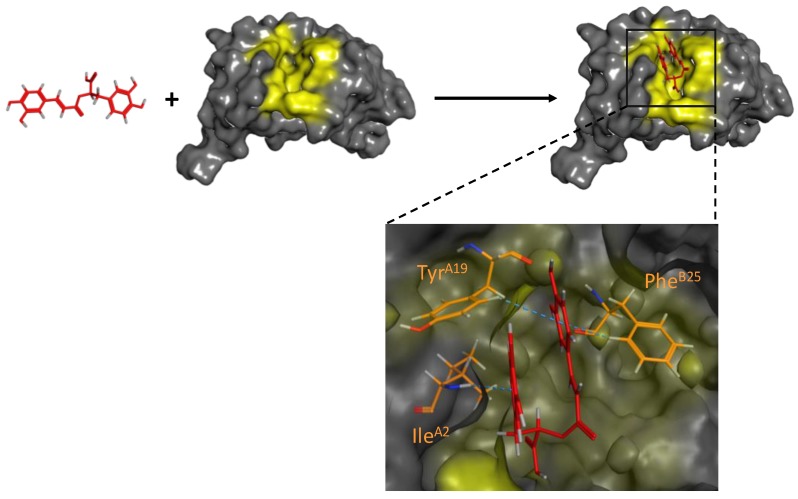
Rosmarinic acid undergoes conformational change from extended to bent conformation in binding to the hydrophobic pocket in insulin dimer. The formation of an aromatic cluster between bent rosmarinic acid and aromatic residues on the surface stabilizes the dimer, precluding amyloid formation.

Recently, we used a combination of solution-state NMR and molecular docking to elucidate the mechanism of the inhibition of insulin amyloid formation by RA [15]. Insulin is an attractive model protein for amyloid self-assembly because the 3D structures of insulin oligomers are known. Our results show that RA binds to a hydrophobic pocket in insulin dimer and in doing so, the polyphenol undergoes a conformational change from an extended structure to a bent conformation (Figure 5). Importantly, the aromatic moieties of the polyphenol form π-π interactions with aromatic residues in the pocket to form an extended aromatic network, resulting in inhibition of amyloid formation [15]. Our work suggests that polyphenols that have the ability to form extended aromatic clusters with aromatic residues on the surface of an amyloidogenic protein has a high potential to be an effective inhibitor.

CUR is a potent inhibitor of Aβ42 oligomerization [51,52]. Yang and coworkers dissolved Aβ42 in hexafluoroisopropanol (HFIP) to prepare seedless Aβ42 monomers. After removal of HFIP, they then used the oligomer preparation protocol reported by Kayed et al. [53] to test the ability of CUR to inhibit oligomerization. Dot blots indicated dose-dependent inhibition of Aβ42 oligomerization [51]. Subsequent work by Reinke and Gestwicki identified the contribution of each structural module in CUR to inhibition of Aβ42 oligomerization [52]. CUR contains two relatively polar aromatic groups joined by a rigid linker (Table 1). Both polar groups at each end of the molecule and the hydroxy substitutions on them are required for inhibition. The optimal length of the linker lies between 8 Å and 16 Å [52]. We noted that RA meets these requirements, providing support to the structure-activity relationships obtained by Reinke and Gestwicki.

#### 3.2.3. Remodeling of Aβ Oligomers to Nontoxic Forms

Ehrnhoefer et al. used biochemical and cell biological methods to study the effect of EGCG on the oligomerization of Aβ42 [54]. EGCG does not inhibit oligomerization but the oligomers formed are off-pathway (i.e., they are structurally distinct from Aβ42 amyloid oligomers), seeding incompetent, and are nontoxic to mammalian cells by the standardized 3-(4,6-dimethylthiazol-2yl)-2,5-diphenyltetrazolium bromide reduction assay. The EGCG-induced amelioration of toxicity was also observed in larger assemblies of Aβ42. Using cell-free and cell-based assays, Bieschke et al. showed that EGCG binds to large oligomers and preformed fibrils and remodels them into less toxic off-pathway assemblies [55]. These results have led to experimental and computational studies of the structure of the complexes formed by EGCG and Aβ [54,56,57,58,59]. Ehrnhoefer et al. showed that EGCG induces formation of spherical Aβ42 assemblies that are nonamyloidogenic [54]. Analysis of two-dimensional magic-angle spinning solid-state NMR correlation spectra of EGCG-induced Aβ40 oligomers indicated that the polyphenol interferes with the aromatic core region (i.e., residues 10–20) of Aβ40 [56]. More recently, Ahmed et al. used solution-state NMR, dynamic light scattering and electron microscopy to investigate how EGCG remodels Aβ40 oligomers in solution [57]. They showed that the remodeling adheres to a Hill-Scatchard model, i.e., EGCG binds to equivalent and independent sites within Aβ40 oligomers. Upon binding EGCG, the oligomers become less exposed to solvent and the Aβ monomer-Aβ oligomer contacts become less engaged. The authors concluded that EGCG inhibits the secondary nucleation events that generate toxic Aβ oligomers [57]. An all-atom molecular dynamics simulation by Zhang et al. showed that Aβ42 dimers in the presence of EGCG adopt new conformations characterized by increased α-helix and unstructured contents at the expense of β-sheet, reduced intra- and interchain contacts, and increased inter-center-of-mass distances [59]. However, Nguyen and Derreumaux in a recent overview of Aβ oligomer—drug interactions from computer simulations noted that simulations of Aβ42–EGCG complexes show that there is room for a more potent inhibitor that would bind more tightly and sequester Aβ42 dimers from Aβ42 monomer more efficiently [58].

RES does not inhibit Aβ42 oligomerization [60,61,62], presumably because it lacks the structural features common to CUR and RA (vide supra). Nonetheless, Feng et al. showed that RES attenuates the cytotoxicity of Aβ42 oligomers presumably by remodeling the oligomers into nontoxic conformers [60]. The capacity of RES to remodel Aβ42 assemblies was also reported by Ladiwala and coworkers [61]. They noted that RES remodels Aβ42 soluble oligomers, fibrillar intermediates and amyloid fibrils into aggregates that are negative for multiple conformational probes (e.g., conformation-specific antibodies and ThT) and nontoxic. Structural details of the interaction of RES with Aβ42 oligomers were investigated by Fu and coworkers using solution-state NMR and atomic force microscopy [62]. RES binds to the N-terminus of Aβ42 and limits oligomer formation to low molecular weight oligomers. This result suggests that the N-terminus of Aβ42 plays a key role in the formation of high molecular weight oligomers.

## 4. Polyphenols Inhibit Tau Self-Assembly

### 4.1. Polyphenols Modulate Tau Hyperphosphorylation

**Figure 6:**
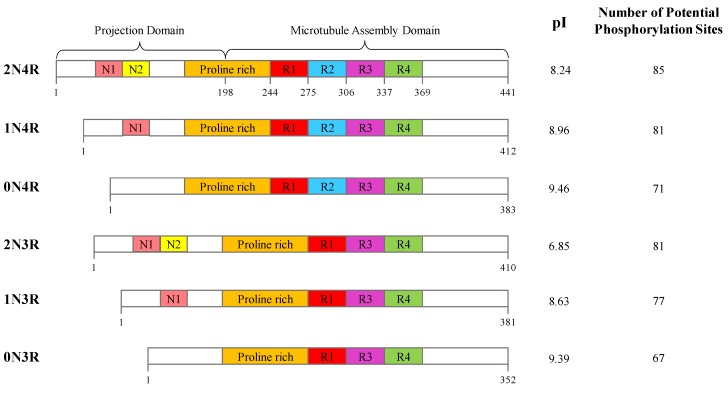
Tau isoforms in the human brain. Tau consists of a projection domain and a microtubule-assembly domain. The six tau isoforms (2N4R, 1N4R, 0N4R, 2N3R, 1N3R, AND 0N3R) are designated by the number of N inserts in the former and by the number of repeats R in the latter. Calculated pI’s indicate that the isoforms except 2N3R are basic proteins. The number of potential phosphorylation sites in each isoform is indicated.

The human brain contains six major tau isoforms: 2N4R, 1N4R, 0N4R, 2N3R, 1N3R, AND 0N3R (Figure 6). These isoforms differ in the number of inserts N near the N-terminus, which can be 0, 1, or 2, and in the number of microtubule-binding repeats R, which can be 3 (i.e., R2 is missing) or 4. Each isoform contains two domains: a projection domain that extends from the surface of microtubules and a microtubule-binding domain. Calculated pI’s indicate that the tau isoforms with the exception of 2N3R, are basic proteins (Figure 6). The dominance of repulsive positive charges may account for the absence of tau self-assembly in pure buffer. A common posttranslational modification that may facilitate tau self-assembly is phosphorylation. Because tau in NFTs is hyperphosphorylated [63], phosphorylation has been assumed to trigger self-assembly. This makes sense because abnormal hyperphosphorylation at several sites may compensate for the repulsive positive charges in tau [64]. However, because in vitro studies have shown that tau aggregation can be induced by the presence of polyanionic cofactors [65], some of which could be present in vivo, the importance of phosphorylation in tau self-assembly remains a matter of debate. This is complicated by the large number of potential phosphorylation sites in tau, which ranges from 67 in 0N3R, the shortest tau isoform, to 85 in 2N4R, the longest tau isoform (Figure 6), and by the diversity in their locations in the molecule. Nonetheless, hyperphosphorylation of tau may lead to disease through other mechanisms. Dissociation of hyperphosphorylated tau from mictrobules may result in the breakdown of the microtubular cytoskeleton [66]. Hyperphosphorylation of tau may induce tau mislocalization, which in turn can lead to synaptic dysfunction [67]. Phosphorylation at specific sites may diminish the degradation of tau [68], which can then lead to increased levels of tau favoring self-assembly.

**Figure 7:**
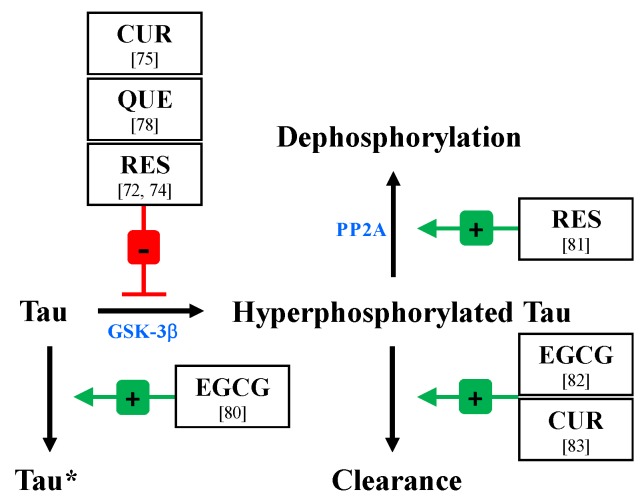
Polyphenols modulate levels of hyperphosphorylated tau by inhibiting GSK-3β activity towards tau, remodeling tau to kinase-resistant tau (tau*), increasing the activity of PP2A towards hyperphosphorylated tau, and enhancing the clearance of hyperphosphorylated tau.

The degree of phosphorylation of tau in neuronal cells is regulated by the balancing act of phosphatases and serine/threonine kinases. The major phosphatase and kinase for neuronal tau are phosphatase 2A (PP2A) [69] and glycogen synthase kinase 3β (GSK-3β) [70,71], respectively. Figure 7 presents four ways by which polyphenols modulate tau hyperphosphorylation: (1) inhibition of the activity of GSK-3β towards tau; (2) remodeling tau to tau*, i.e., tau resistant to kinase action; (3) increasing the activity of PP2A towards hyperphosphorylated tau; and (4) enhancing the clearance of hyperphosphorylated tau.

#### 4.1.1. Inhibition of GSK-3β and Other Kinases

He et al. showed that RES inhibits the formaldehyde-induced hyperphosphorylation of tau at Thr181 in a dose-dependent manner [72]. Additional experiments showed that the inhibition results from the suppression of the catalytic activities of GSK-3β and calmodulin-dependent protein kinase II (CaMKII), another kinase implicated in tau hyperphosphorylation. In senescence accelerated mice P8 (SAMP8), a neuropathological model of accelerated brain aging and dementia [73], RES inhibits the activity of cyclin-dependent kinase 5 and GSK-3β, preventing tau phosphorylation at Ser396 [74]. Wang et al. showed that exosomes derived from CUR-treated cells (Exo-cur) inhibit the hyperphosphorylation of tau through the AKT/GSK-3β pathway in an animal model of AD generated by injecting okadaic acid in the brain of C57BL/6 mice [75]. Okadaic acid induces the hyperphosphorylation of tau [76,77] by inhibiting PP2A [75]. Jiang et al. investigated the neuroprotective effects of QUE against okadaic acid-induced toxicity in HT22 cells obtained from mouse hippocampal tissue [78]. Okadaic acid induced tau hyperphosphorylation at Ser199, Ser396, Thr205 and Thr231 and oxidative stress in the HT22 cells. However, treatment with QUE prevented oxidative stress and tau hyperphosphorylation by inhibition of the PI3K/AKT/GSK-3β signaling pathway. Together, these studies indicate that polyphenols have the potential of inhibiting kinases implicated in the hyperphosphorylation of tau. While it could be true that the modulation of kinases is likely to affect other key pathways, we surmise that if modulation of kinase activity is to be targeted, then the use of polyphenols would be an attractive approach due in part to other additional health benefits these molecules provide.

#### 4.1.2. Remodeling of Tau to Tau*

Guo et al. reported that long-term oral consumption of EGCG ameliorated the impaired working memory and spatial learning memory in SAMP8 mice, determined by Y-maze and Morris water maze tests, respectively [79]. In addition to a reduction of Aβ42 levels, EGCG treatment also prevented tau hyperphosphorylation. To obtain molecular and structural insights into the inhibition of tau phosphorylation by EGCG, Guéroux et al. studied the proline-rich region (PRR) of 2N4R tau where most of the phosphorylation sites are located [80]. Two peptides were synthesized, one corresponds to Ile171–Lys190, the first PRR of tau, and the other corresponds to Ile171–Thr220, which contains more than 50% of the PRR of tau. Using a combination of NMR and molecular modeling, they showed that EGCG modifies the 3D structures of the peptides and binds to the putative phosphorylation sites such that access by kinases is diminished [80].

#### 4.1.3. Enhancement of PP2A Activity

Another mechanism that has been proposed for RES also modulates the levels of hyperphosphorylated tau by increasing tau dephosphorylatation. Schweiger et al. showed that the polyphenol significantly increases the activity of PP2A [81]. The enhancement of the activity of PP2A is caused by decreased expression of MID1 ubiquitin ligase that facilitates the degradation of the catalytic subunit of PP2A. Intriguingly, Schweiger et al. also showed that MID1 expression is increased in AD tissue [81].

#### 4.1.4. Increased Clearance of Phosphorylated Tau

EGCG and CUR have been shown to facilitate the clearance of hyperphosphorylated tau. Chesser et al. demonstrated that EGCG has the ability to enhance the clearance of phosphorylated tau [82] by increasing mRNA expression of two key autophagy adaptor proteins, NDP52 and p62. Ma et al. [83] used wild-type human tau transgenic mice, which exhibits established tau pathology and neuron loss [84], to determine the effect of CUR on tau-induced synaptic and cognitive deficits. Mice were fed PMI 5015 with 500 ppm CUR, a formulation that has shown to increased levels of bioavailable free CUR in the brain [85]. Behavioral and cognitive tests on the mice showed that the polyphenol corrected tau-dependent behavioral and synaptic deficits. To elucidate the mechanism behind these results, Ma et al. examined hippocampal tissue and found that CUR elevated levels of heat shock proteins involved in the clearance of phosphorylated tau dimers [83], which have been hypothesized to play a critical role in cognitive and synaptic dysfunction [86].

### 4.2. Polyphenols Inhibit Tau β-Sheet Formation

The self-assembly of tau shares three common features with the self-assembly of Aβ. First, tau undergoes a random coil → β-sheet conformational rearrangement [87,88]. Tau in solution is predominantly unstructured as revealed primarily by circular dichroism (CD) spectroscopy [89]. As noted above, tau in pure buffer does not self-assemble but in the presence of polyanionic species, self-assembly takes place. Goedert et al. showed that incubation of tau with heparin at 37 °C leads to the formation of Alzheimer-like tau filaments [90]. Other negatively charged molecules including RNA [91] and free fatty acids such as arachidonic acid [92] also facilitate tau self-assembly to filaments. Together, these studies indicate that tau self-assembly to filaments is driven primarily by electrostatics rather than by the precise structure of the negatively charged species. Berriman et al. used X-ray diffraction (XRD) and Fourier transform infrared spectroscopy (FTIR) to show that tau filaments contain cross-β structure [93]. Solid-state NMR [94], XRD and solution-state spectroscopic analysis by CD and FTIR [95] of filaments formed by tau peptides (vide infra) also reveal the presence of β-sheet.

**Figure 8:**
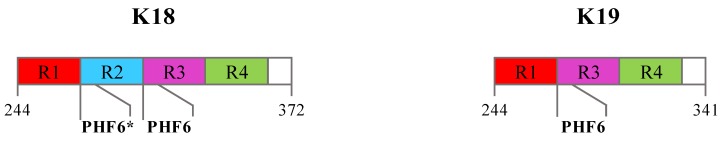
Tau constructs that form filaments. K18 and K19 correspond to the repeat domains of 2N4R and 0N3R, respectively. The relative locations of the hexapeptide motifs (PHF6* (VQIINK) and PHF6 (VQIVYK)), which are thought to act as nucleating segments for tau self-assembly are indicated.

Second, fragments of tau also self-assemble to form filaments. When tau self-assembles to PHFs, the repeat regions (R1 to R4) form the core, while the long N-terminal and shorter C-terminal domains surround the core [96]. The dominant secondary structure present in the core of PHFs is β-sheet while the N- and C-terminal domains projecting from the core are predominantly random coil [97]. Together, these results suggest that peptides corresponding to the repeat regions of tau will self-assemble to filaments similar to those formed by full-length tau. Indeed, K18, which corresponds to R1-R4 in 2N4R, and K19, which corresponds to R1, R3 and R4 in 0N3R (Figure 8), form filaments similar to those formed by full-length tau isoforms but self-assemble more aggressively presumably because they do not contain the N- and C-terminal domains that modulate intermolecular interactions involving the repeat regions [98,99].

Last, tau self-assembly is also inhibited by polyphenols. Santa-Maria et al. tested the effect of treating JNPL3 transgenic mice with grape seed polyphenolic extract (GSPE) [100]. JNPL3 mice express human tau containing the P301L mutation [101]. NFTs develop in the brain and spinal cord of the mice, leading to motor and movement abnormalities. Santa-Maria et al. found that GSPE treatment reduced the levels of hyperphosphorylated and sarcosyl-insoluble tau and improved the motor function of the treated mice [100]. The mechanism/s for the effect of GSPE is not well understood. Nonetheless, a polyphenol combination strategy for anti-tau-self-assembly seems to be an attractive approach [13]. Other combinations are possible (e.g., bioactive dietary polyphenol extract [102]) and thus studies that will identify the polyphenol combination that works the best could prove to be useful.

**Figure 9:**
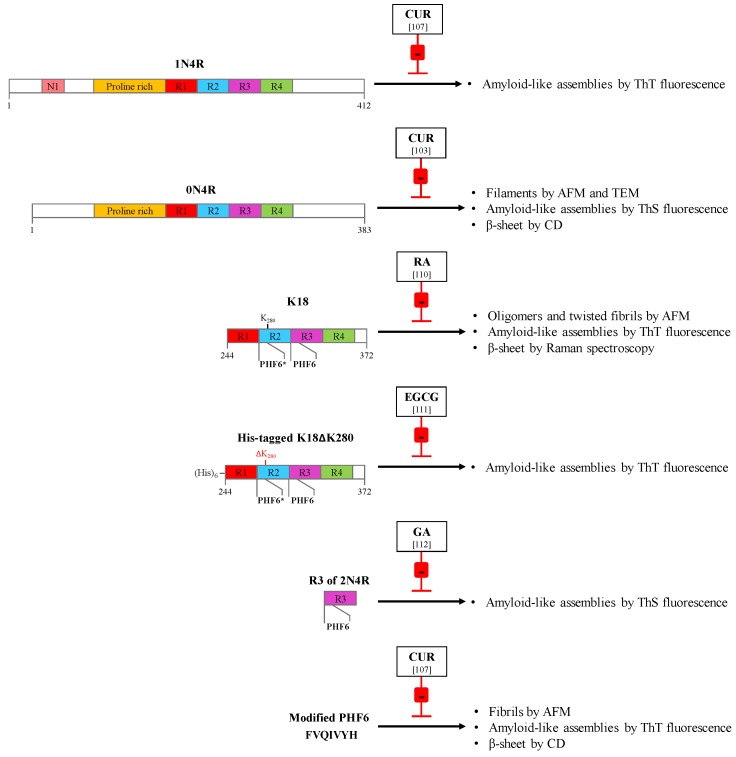
Polyphenols inhibit the self-assembly of full-length tau and fragments.

Biophysical studies of the self-assembly of full-length tau and model peptides in the presence of polyphenols (Figure 9) provide insights into mechanisms of inhibition. Rane et al. showed that 0N4R tau in the presence of arachidonic acid self-assembles to form β-sheet containing filaments [103]. In the presence of CUR, filament formation is abolished. Binding experiments indicated that CUR binds more strongly to 0N4R (K_d_ = 3 μM) than to 0N3R (K_d_ = 8 μM), which lacks R2 (Figure 6). Molecular docking showed that CUR interacts with several residues in the R1–R4 region of 0N4R, including Asp194 and Leu195 in R1, Asp225 in R2, Val255 and Ser258 in R3, and Lys285, Val292 and Val305 in R4, providing a mechanism for the inhibition of β-sheet formation by the polyphenol. Bijari et al. showed through ThT fluorescence, which is sensitive to β-sheet-containing amyloid assemblies [104,105,106], that CUR inhibits the self-assembly of 1N4R tau [107]. Molecular docking revealed that CUR binds to a region close to the nucleating hexapeptide motif designated as PHF6 (V_306_QIVYK_311_) [108] found in R3 of 1N4R [107]. Other naturally occurring polyphenols such as epicatechin 3-gallate and myricetin inhibit the heparin-induced filament formation by 1N4R [109]. Cornejo et al. showed that RA inhibits β-sheet formation by a peptide containing K18 (Figure 8) in a dose-dependent manner [110]. Wobst et al. investigated the ability of EGCG to inhibit the aggregation of His-tagged K18ΔK280, a K18 construct that contains a mutation in R2 linked to frontotemporal dementia (i.e., deletion of Lys280) [111]. Through the use of ThT fluorescence, dot blot analysis using the anti-oligomer antibody A11, and CD spectroscopy, they showed that EGCG inhibits the aggregation of His-tagged K18ΔK280 into toxic oligomers at substoichiometric concentrations. Yao et al. showed that GA inhibits the aggregation of the R3 domain of 2N4R [112]. R3 is a suitable model peptide for the aggregation of full-length tau because its N-terminus contains PHF6 (Figure 9). Interestingly, tannic acid, which is a naturally occurring polymer of GA, is a more potent inhibitor of R3 aggregation [112]. Molecular docking showed that three aromatic rings of tannic acid bind to the PHF6 region. CUR inhibits amyloid formation by the heptapeptide FVQIVYH, which contains the segment VQIVY found in PHF6 [107]. Overall, mechanistic studies of the inhibition of tau self-assembly by polyphenols validate the hypothesis that PHF6 nucleates β-sheet formation in tau.

## 5. Conclusions and Future Directions

The mechanisms presented in this review show that polyphenols inhibit Aβ self-assembly to oligomers, and tau self-assembly to β-sheet assemblies, by affecting both the biochemistry and biophysical chemistry associated with each process. In the case of Aβ self-assembly, polyphenols inhibit oligomerization biochemically by modulating Aβ monomer levels in two ways, i.e., through modification of the activity of secretases associated with the processing of AβPP and enhancement of the clearance of Aβ monomer. Polyphenols inhibit Aβ oligomerization biophysically by interfering with physical contacts between Aβ monomers, which are driven primarily by hydrophobic interactions. In the case of tau self-assembly, polyphenols inhibit this process biochemically through modulation of tau hyperphosphorylation, which is hypothesized to drive aggregation to NFTs. Polyphenols inhibit tau self-assembly biophysically by interfering with the nucleation of β-sheet formation hypothesized to be facilitated by PHF6. Together, the mechanisms presented here underscore the potency of polyphenols to inhibit abnormal self-assembly.

Recent progress in the pathobiology of AD suggests future directions. Self-propagating Aβ species (aka Aβ prions) may play an initiating role in sporadic AD [113]. Can these species be targeted pharmacologically? If so, will polyphenols inhibit their formation and their ability to self-propagate, i.e., to convert a “normal” Aβ species into an additional copy of the Aβ prion? The spread of tau pathology may also occur through prion-like propagation [114]. Can the interneuronal tau propagation be blocked by polyphenols? Finally, cross-seeding of tau self-assembly by aggregated Aβ may account for Aβ-induced propagation of tau pathology [115,116]. Can polyphenols block cross-seeding interactions between Aβ and tau?

AD is a complex disease because many pathological mechanisms are involved including neurodegeneration induced by Aβ self-assembly and neurodegeneration induced by tau self-assembly. Finding a cure for the disease has been elusive. All of the recent therapeutic strategies have targeted Aβ self-assembly and have continued to fail in clinical trials. Therapeutic strategies that simultaneously target Aβ self-assembly and tau self-assembly may lead to better outcomes. Because polyphenols inhibit Aβ self-assembly and tau self-assembly in a number of ways and possess antioxidant and anti-inflammatory properties, the use of naturally occurring polyphenols is an attractive therapeutic approach that should be developed further.

## Figures and Tables

**Scheme 1:** Hierarchical self-assembly of Aβ monomers to fibrils.

Aβ monomers → Aβ oligomers → Aβ Protofibrils → Aβ Fibrils

**Scheme 2:** Hierarchical self-assembly of tau monomers to NFTs.

Unstructured tau monomers → tau oligomers → tau fibrils → PHFs → NFTs

**Table 1:** Structures, common sources and water solubility of polyphenols in this review.

Molecule Common Source/s Solubility in H_2_O	Chemical Structure
**Resveratrol**	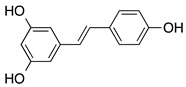
red wine & red grapes	C_14_H_12_O_3_
sparingly soluble	5-[(*E*)-2-(4-hydroxyphenyl)ethenyl]benzene-1,3-diol
**Rosmarinic acid**	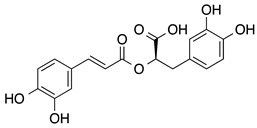
rosemary, basil & sage	C_18_H_16_O_8_
soluble	(2*R*)-3-(3,4-dihydroxyphenyl)-2-[(*E*)-3-(3,4-dihydroxyphenyl)prop-2-enoyl]oxypropanoic acid
**Epigallocatechin-3-gallate**	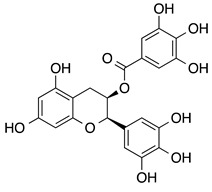
green tea	C_22_H_18_O_11_
slightly soluble	[(2*R*,3*R*)-5,7-dihydroxy-2-(3,4,5-trihydroxyphenyl)-3,4-dihydro-2*H*-chromen-3-yl] 3,4,5-trihydroxybenzoate
**Curcumin**	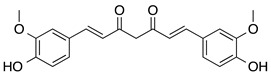
turmeric	C_21_H_20_O_6_
sparingly soluble	(1*E*,6*E*)-1,7-bis(4-hydroxy-3-methoxyphenyl)hepta-1,6-diene-3,5-dione
**Gallic acid**	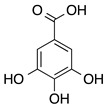
black tea, fruits & nuts	C_7_H_6_O_5_
sparingly soluble	3,4,5-trihydroxybenzoic acid
**Quercetin**	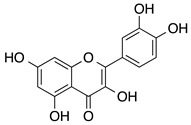
onions, spinach & apples	C_15_H_10_O_7_
sparingly soluble	2-(3,4-dihydroxyphenyl)-3,5,7-trihydroxychromen-4-one
